# Health Tract, No. 237

**Published:** 1866-05

**Authors:** 


HEALTH TRACT, No. 237.

### FOUL ODORS.

It is of vital importance, especially in warm weather, when any disease prevails in a community, to keep the air as pure as practicable; under such circumstances every man owes it to himself and to neighborly comity, to keep his own premises as perfectly clean and pure as possible, and to do this with as little trouble and expense as possible; the following suggestions are made:—A deodorizer simply makes a bad smell imperceptible (see tract 154.) A disinfectant separates the odor into its original elements, and makes new combinations, new substances, which are hurtless. Fresh burned lime, called unslacked or quick lime, is the most common disinfectant, dissolved in water until it is thin enough to be sprinkled, or used with a brush.

Copperas, called green vitriol or sulphate of iron, is better than lime; a pound costing half a dime, dissolved in four gallons boiling water, and thrown into a privy or sink, will remove the odor in ten minutes, to be repeated two or three times a month in warm weather, or as often as any odor is perceptible.

Hydrated per-chloride of Iron, i. e. Copperas roasted and made into a paste, one pint to ten pints of water, is perhaps the most efficient deodorizer and disinfectant known.

Chloride of Lime, sprinkled over damp places, in yards, cellars &c., cleanses and purifies, but it has an odor of its own and is supposed by some to be hurtful.

A layer of fresh burned charcoal in powder, two or three inches in depth, over a heep of decaying offal, absorbs the odor, decomposes it and burns it up.

If this cannot be had, a layer of fresh earth, six inches deep, confines the odor, of decaying matters, but does not destroy them, and is good for temporary use until better substances can be obtained. Sulphurated Hydrogen has an odor similar to that of rotten eggs; it arises from sinks, privies, and heaps of decaying animal and vegetable matters—it is that which blackens our door plates; it is effectually decomposedand destroyed by the Hydrated Per-chloride of Iron.

The manganates of soda or potash, dissolved in warm water are among the best deodorisers and disinfectants.

To disinfect linen, or washing apparel, soak it in a mixture of one ounce of Chloride of Lime, in a gallon of water.

Woolens, bedding, &c., which cannot be washed, are best disinfected by exposure for three or four hours in a chamber, heated to two hundred degrees.

To disinfect rooms, wash the ceilings and walls with quicklime water, and scrub the woodwork with brush, soap, and hotwater, and then wash with two ounces of Chloride of Lime, dissolved in a gallon of water.

Scientific experiments seem to indicate that great good results in rooms, where there is small pox and other diseases giving out organic poisons, by putting some Iodine in a box with a lid full of holes, the fumes soon pervade the room, giving a violet tinge to some household implements.

Glass and stoneware after being scrubbed with sand and soap are deprived of all ill odor by shaking dry charcoal powder in them.

